# Impact of anti-HER2 therapy on overall survival in HER2-overexpressing breast cancer patients with brain metastases

**DOI:** 10.1038/bjc.2011.531

**Published:** 2011-11-29

**Authors:** R Bartsch, A Berghoff, U Pluschnig, Z Bago-Horvath, P Dubsky, A Rottenfusser, C DeVries, M Rudas, F Fitzal, K Dieckmann, R M Mader, M Gnant, C C Zielinski, G G Steger

**Affiliations:** 1 Clinical Division of Oncology, Department of Medicine I, Medical University of Vienna, Waehringer Guertel 18-20, Vienna 1090, Austria; 2 Comprehensive Cancer Centre, Medical University of Vienna, Waehringer Guertel 18-20, Vienna 1090, Austria; 3 Department of Pathology, Medical University of Vienna, Waehringer Guertel 18-20, Vienna 1090, Austria; 4 Department of Surgery, Medical University of Vienna, Waehringer Guertel 18-20, Vienna 1090, Austria; 5 Department of Radiotherapy, Medical University of Vienna, Waehringer Guertel 18-20, Vienna 1090, Austria

**Keywords:** brain metastases, breast cancer, HER2-positive disease, lapatinib, trastuzumab

## Abstract

**Background:**

Trastuzumab-based therapy after diagnosis of brain metastases (BM) may improve survival due to prolonged systemic disease control. We investigated whether lapatinib may yield additional survival benefit.

**Methods:**

Eighty patients with BM from HER2-positive breast cancer were identified. Karnofsky Performance Score (KPS) of at least 70 was required. We included a control group of 37 patients treated before 2003, when continuation of trastuzumab after diagnosis of BM was not yet recommended. Remainders received either trastuzumab or lapatinib and trastuzumab (either concomitantly or sequentially) with or without chemotherapy.

**Results:**

Median overall survival (OS) in patients receiving trastuzumab after diagnosis of BM was 13 months; corresponding numbers were 9 months in patients treated with chemotherapy, and 3 months with radiotherapy alone. Median OS was not reached in the lapatinib group. Addition of lapatinib prolonged OS over trastuzumab alone (*P*=0.002). After correction for potential confounders, lapatinib therapy remained an independent positive predictor for survival (HR 0.279; *P*=0.012).

**Interpretation:**

This retrospective single-centre study suggests that the introduction of lapatinib improved survival in patients with BM from HER2-positive breast cancer. Patients with KPS ⩾70 may benefit when treated with lapatinib in addition to trastuzumab after completion of local therapy.

Among solid cancers, breast cancer is the second most common cause of central nervous system (CNS) metastases and the most common cause of carcinomatous meningitis (Weil *et al*, 2005; Stemmler and Heinemann, 2008). Various risk factors were identified: young age, advanced and hormone receptor-negative disease, short disease-free interval, high disease burden, and visceral metastases (Miller *et al*, 2003; Evans *et al*, 2004; Lin *et al*, 2004; Slimane *et al*, 2004; Weil *et al*, 2005). A marked increase in the incidence of brain metastases (BM) was observed in HER2-positive patients over the last decade. This finding is usually attributed to the survival benefit associated with the introduction of trastuzumab, a monoclonal antibody targeting HER2; furthermore, trastuzumab, due to its molecular size, has limited ability to pass through the blood–brain barrier, rendering the CNS an important tumour cell sanctuary (Bendell *et al*, 2003; Clayton *et al*, 2004; Shmueli *et al*, 2004; Burstein *et al*, 2005; Viani *et al*, 2007). In favour of that notion, the rate of patients with brain as first site of disease progression is increasing by time (Yau *et al*, 2006).

Treatment for BM consists of corticosteroids, whole brain radiotherapy (WBRT) as well as neurosurgical resection, radiosurgery, and boost irradiation as indicated (Borgelt *et al*, 1980; Bindal *et al*, 1993; Lohr *et al*, 2001). Whole brain radiotherapy yields symptomatic and clinical responses in ∼50% of patients, while survival remains dismal at 6 months (Lutterbach *et al*, 2002; Broadbent *et al*, 2004). Systemic therapy has limited impact on BM (Rosner *et al*, 1986). While three recent studies reported better survival outcomes when patients with BM received further trastuzumab after completion of local therapy, it is assumed that the impact on overall survival (OS) is due to control of systemic disease rather than brain lesions (Lower *et al*, 2003; Kirsch *et al*, 2005; Bartsch *et al*, 2007). Lapatinib, a small molecule tyrosine-kinase inhibitor of EGFR and HER2, was recently approved for the treatment of HER2-positive metastatic breast cancer. Due to its small molecular size, lapatinib may pass the blood – brain barrier, opening possibilities for medical treatment and prophylaxis of CNS metastases (Cameron *et al*, 2008; Lin *et al*, 2008). Indeed, two phase II studies conducted in patients with established BM reported a modest yet significant activity of lapatinib by indicating a volumetric reduction in the size of brain lesions (Lin *et al*, 2008, 2009). Importantly, the 2-year OS was higher in patients with BM responding to lapatinib-based therapy as compared with those with stable or progressive CNS disease (66% *vs* 44%). This suggests that with improved systemic disease control, better local control of brain lesions yields additional survival benefit.

Based upon those assumptions, we investigated whether lapatinib-based treatment may improve survival outcome in patients with BM from HER2-positive breast cancer. Accordingly, we compared patients receiving lapatinib and trastuzumab (either sequentially or concomitantly) after completion of local therapy with individuals who only received trastuzumab plus/minus chemotherapy and a historical control group of HER2-positive subjects without any further targeted therapy.

## Patients and methods

Patient data were collected at the Comprehensive Cancer Centre, Medical University of Vienna. This retrospective analysis was approved by the local ethics committee.

### Patients

**Figure 1:**
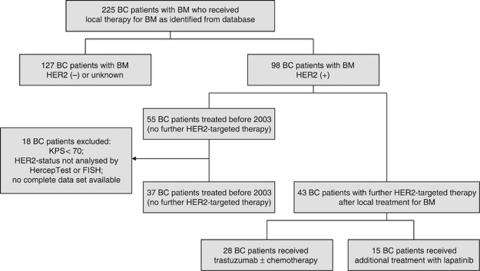
Patient cohort. BC=breast cancer; BM=brain metastases; HER2 (+)=HER2 positive; HER2 (−)=HER2 negative.

Data from all consecutive patients who were treated with local therapy for BM from HER2-positive breast cancer from 2003 until 2010 who received trastuzumab and/or lapatinib after completion of local therapy for BM were retrieved from a breast cancer database (group A). Patients without further systemic therapy or Karnofsky Performance Score (KPS) <70 were not included to avoid an inclusion bias, as low KPS is a known negative predictor of OS. In a second step, data were retrieved from patients who received local treatment for BM between 1998 and 2002, and served as control; 2002 was chosen as cutoff, as from 2003 onwards continuation of trastuzumab treatment after diagnosis of BM was generally recommended. Again, patients with KPS <70 or incomplete data sets were excluded (group B). Within group B, patients either received chemotherapy after completion of local treatment or no further systemic therapy at all. This decision was taken at the discretion of the treating physician and patients without further chemotherapy were thought to have no meaningful systemic treatment option left. In total, 80 patients were available for this retrospective analysis (Figure 1).

### Treatment plan and patient evaluation

In patients with >3 metastases, WBRT was applied at a 6-MV linear accelerator (LINAC) by lateral opposed fields. Total dose prescribed was 30 Gray (Gy) in 10 fractions of 3 Gy. In case of one to three metastases ⩽2 cm, a stereotactic boost was applied at a Gamma knife (16–20 Gy on the 50% isodose), or at a 6-MV LINAC (20 Gy on the 80% isodose). In case of tumour size >2 cm, two times 10 Gy were applied at a 6-MV LINAC. Boost irradiation was applied either alone or in combination with WBRT. In selected cases, prior neurosurgical resection had been performed.

Trastuzumab was administered at a dose of 6 mg kg^−1^ body weight every 3 weeks after a loading dose of 8 mg kg^−1^ body weight on the first day of treatment. Lapatinib was administered at a daily fixed dose of 1000 mg (in combination with trastuzumab), 1250 mg (in combination with capecitabine), or 1500 mg (as single agent) with appropriate dose reductions if necessary.

HER2 status was assessed using the HercepTest (Dako A/S, Glostrup, Denmark) or dual colour fluorescent *in-situ* hybridisation (FISH; PathVision HER2 DNA probe kit, Vysis Inc., Downers Grove, IL, USA). Tumours were classified as HER2 positive if they had a staining intensity of +++ on the HercepTest; if a score of ++ was gained, tumours were reanalysed by FISH. In patients with neurosurgical resection of BM, HER2 status was reassessed from the CNS lesion. From 2008 onwards, HER2 status was generally reassessed at the time of first diagnosis of metastatic disease. In those patients, a biopsy from one metastatic site was taken whenever feasible. In patients progressing before 2008, HER2 status was not reassessed and HER2 status of the primary tumour was used as surrogate.

Oestrogen and progesterone receptor status were assessed by immunohistochemistry (ER*α* antibody, clone 1D5; Dako A/S and PgR antibody; Dako A/S). Receptor expression was estimated as the percentage of positively stained tumour cells. Results were given as +, ++, +++ positive staining or negative staining, with a cutoff value of <10% positive tumour cells.

All patients had symptomatic BM and no routine screening for CNS involvement was conducted. For baseline staging evaluations, all patients had CT scans of the chest and abdomen, mammography, and gynaecologic examination. Before initiation of trastuzumab or lapatinib treatment, echocardiography was mandatory, and patients with left ventricular ejection fraction (LVEF) <50% were excluded. Restaging was performed every 3 months. Cranial MRI scans were performed 1 and 3 months after completion of local therapy, and every 3 months thereafter. In patients treated before 2003, cranial CT scans instead of MRI scans were repeatedly performed during follow-up, thus complicating the assessment of response and progression-free survival (PFS) within the CNS. Furthermore, restaging was not always conducted at regular 12-weekly intervals; therefore, no analysis of CNS response and PFS was performed.

### Statistical analysis

Overall survival was chosen as primary study end point; OS was defined as interval from diagnosis of symptomatic BM until death of any cause and estimated using the Kaplan–Meier product limit method. To test for differences between respective OS curves, the log-rank test was used. *P*-values <0.05 were considered to indicate statistical significance.

The following variables were included in the univariate analysis of OS: type of systemic therapy after completion of local treatment (no further treatment, chemotherapy only, any treatment including trastuzumab, any treatment including lapatinib); pathological subtype (ductal *vs* lobular); grading (grade 1 and 2 *vs* 3); hormone receptor status; presence of visceral metastases; brain as only site of metastatic disease; presence of >2 metastatic sites outside the CNS; early diagnosis of BM (<12 months from diagnosis of breast cancer or metastatic disease); age >65 years at diagnosis of BM; KPS (70 *vs* >70); number of brain lesions (1–3 *vs* >3).

A Cox proportional hazard model was used to correct for factors significantly (*P*<0.05) or borderline significantly (*P*<0.07) associated with OS on the univariate analysis.

Differences between demographic data are described with frequencies and percentages and were tested with the Fisher's exact test.

Data were analysed as of February 2011. All statistics were calculated using statistical package for the social sciences (SPSS) 17.0 software (SPSS Inc., Chicago, IL, USA).

## Results

### Patient characteristics

Overall, 80 patients with KPS >70 treated for BM from HER2-positive breast cancer were identified from a breast cancer database. Forty-three patients received anti-HER2 treatment after completion of local therapy (group A). Within this cohort, 28 patients received trastuzumab plus/minus chemotherapy and another 15 were additionally treated with lapatinib. A total of 37 patients who were treated before 2003 and therefore received no further anti-HER2 treatment after completion of local therapy were available as control group for this analysis (group B). Within this group, 9 patients received chemotherapy while 28 had no further systemic treatment.

In the total sample of 80 patients, median age at diagnosis of BM was 53 years (range 28–77 years), and 14 patients were >65 years. Median KPS was 80 (range 70–100); 62 patients had KPS of >70. Median time to diagnosis of BM from diagnosis of breast cancer was 36 months (range 0–254 months). In 9 patients, brain was the only site of metastatic disease, 56 had visceral metastases at the time BM were diagnosed, and 39 patients presented with more than two metastatic sites. Seventy-three patients received WBRT, with the remainders treated with boost irradiation only. An additional neurosurgical resection was conducted in 15 subjects. Median duration of trastuzumab therapy after completion of local therapy was 8.5 months, and median duration of lapatinib-based therapy was 8 months. Characteristics of the total patient sample are summarised in Table 1.

The only imbalance between both treatment groups (group A (further anti-HER2 therapy) and group B (no further anti-HER2 therapy)) was the percentage of patients treated with boost irradiation or radiosurgery alone without WBRT as local therapy for BM: 1 out of 37 (2.7%) in the years before 2003 *vs* 6 out of 43 (14%) thereafter (n.s.). This difference likely demonstrates a change in treatment routine and is unlikely to impact on survival. Patient characteristics separated for groups A and B are summarised in Table 2.

### Outcome

Median OS in the total population of 80 patients (groups A and B combined) was 10 months (95% CI: 6.31–13.69).

**Figure 2:**
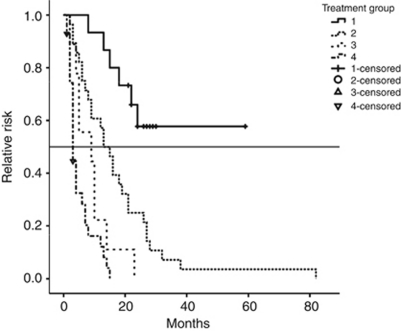
OS (overall survival) after local treatment for brain metastases in all patients (months). Group 1: Patients who received lapatinib and trastuzumab with or without chemotherapy after local treatment for brain metastases, *n*=15. Group 2: Patients who received trastuzumab with or without chemotherapy after local treatment for brain metastases, *n*=28. Group 3: Patients who received chemotherapy without anti-HER2 therapy after local treatment for brain metastases, *n*=9. Group 4: Patients without further systemic therapy after local treatment for brain metastases, *n*=28.

Subgroup analysis: In patients receiving trastuzumab after completion of local therapy for BM, median OS was 13 months (95% CI: 8.85–17.15). Corresponding numbers were 9 months (95% CI: 0–20.69) in patients treated with chemotherapy, and 3 months (95% CI: 2.37–3.63) in patients with radiotherapy alone, respectively. After a median follow-up of 24 months (range 8–46 months), median OS was not reached in the lapatinib group (Figure 2).

In the univariate model, trastuzumab after completion of local therapy resulted in a significant prolongation of OS compared with the control group of 37 patients without anti-HER2 targeted treatment after radiotherapy (*P*<0.001). Addition of lapatinib again led to a significant prolongation of survival over trastuzumab-based treatment plus/minus chemotherapy (*P*=0.002).

Other factors associated with OS in the entire population of all 80 patients (groups A and B combined) included: hormone receptor status (median OS hormone receptor-negative 8 months *vs* 13 months in hormone receptor-positive patients; *P*=0.033); presence of visceral metastases (median OS in patients with visceral metastases 9 months *vs* 12 months in those without; *P*=0.011); early development of BM (i.e., within 12 months from primary diagnosis of breast cancer or diagnosis of metastatic disease *vs* later) (median OS in patients with early development of BM 6 months *vs* 12 months; *P*=0.057); KPS (median OS KPS >70 13 months *vs* 4 months in patients with KPS=70; *P*=0.001); presence of one to three BM (median OS 18 months *vs* 4 months in patients with >3 BM; *P*<0.001).

After correction for those factors, anti-HER2 targeted therapy remained a highly significant predictor for longer OS in the Cox regression model of all 80 patients (HR 0.29; 95% CI: 0.16–0.54; *P*<0.001). Presence of one to three BM (HR 0.32; 95% CI: 0.18–0.58; *P*<0.001) as well as higher KPS (HR 0.4; 95% CI: 0.23–0.72; *P*=0.002) remained also significantly associated with better survival in the multivariate comparisons (Table 2).

In patients treated with anti-HER2 targeted therapy after completion of local treatment (*n*=43; group A alone), median OS was 18 months (95% CI: 12.49–23.51). Factors associated with OS in the univariate analysis included: Presence of visceral metastases (median OS 15 *vs* 32 months; *P*<0.001); more than two metastatic sites (median OS 15 *vs* 21 months; *P*=0.017); KPS (median OS KPS >70 21 months *vs* 7 months; *P*=0.015); presence of one to three BM (median OS 22 *vs* 8 months; *P*<0.001).

After correction for those factors, additional treatment with lapatinib after completion of local therapy remained a significant predictor of longer OS (HR 0.279; 95% CI: 0.1–0.76; *P*=0.012). Again, presence of one to three BM was also significantly associated with superior outcomes (HR 0.152; 95% CI: 0.06–0.41; *P*<0.001; Table 3).

## Discussion

Breast cancer is currently the second most common cause of CNS metastases among all solid cancers. Therefore, BM represent an important cause of morbidity and mortality in breast cancer patients (Lin and Winer, 2007). An increase in the incidence of BM was observed in recent years, which is usually attributed to the introduction of trastuzumab-based therapy (Bendell *et al*, 2003; Clayton *et al*, 2004; Shmueli *et al*, 2004; Burstein *et al*, 2005; Viani *et al*, 2007). Recently, it was reported that up to 40% of all HER2-positive metastatic breast cancer patients will be eventually diagnosed with BM (Bartsch *et al*, 2009). Other studies, however, suggest that even in the absence of trastuzumab, women with HER2-positive metastatic breast cancer are at increased risk for BM as compared with women with HER2-negative disease (Pestalozzi and Brignoli, 2000; Pestalozzi *et al*, 2006). Those divergent results may be explained by the fact that HER2-positive disease has a natural propensity to BM; that effect cannot be fully overcome by trastuzumab, as the antibody, due to its large molecular size, cannot pass through an intact blood–brain barrier and therefore does not offer potential for prophylaxis (Pestalozzi and Brignoli, 2000; Stemmler *et al*, 2007). Under conditions of an impaired blood–brain barrier such as carcinomatous meningitis or radiotherapy, however, trastuzumab levels within the cerebrospinal fluid are increased, thus serving as rational for continuing trastuzumab after diagnosis of BM (Stemmler *et al*, 2007).

BM are usually diagnosed relatively late in the course of metastatic breast cancer; therefore, they commonly occur in patients with advanced systemic disease, as shown in this study. For that reason, it was observed that most patients with BM are in fact dying from systemic disease progression as specific cause of cancer death rather than from BM (Engel *et al*, 2003). This assumption is strengthened by data from two observations showing that in the absence of trastuzumab after diagnosis of brain metastasis, shorter survival results in HER2-positive as compared with HER2-negative patients, again highlighting the intrinsic aggressiveness of the HER2-positive phenotype and the importance of systemic disease control (Kirsch *et al*, 2005; Tham *et al*, 2006).

Since the introduction of trastuzumab, however, various publications reported superior outcomes in terms of OS in patients receiving further systemic therapy, particularly when trastuzumab was included (Bartsch *et al*, 2007; Dawood *et al*, 2008; Park *et al*, 2009). As no significant impact of trastuzumab on the progression of BM could be identified, we suggest that the main impact of trastuzumab on survival results from improved systemic disease control. Indeed, continuation of trastuzumab exhibits activity even in patients progressing during prior trastuzumab-based therapies (Von Minckwitz *et al*, 2009). In line with this assumption, lack of systemic disease control was obviously the major limiting factor for survival of women before the trastuzumab era, as the proportion of patients dying from brain lesions instead of systemic disease progression has increased since the introduction of trastuzumab (Kirsch *et al*, 2005; Lin and Winer, 2007). This points to the urgent need to develop further systemic treatment options exhibiting increased activity in CNS metastases.

Lapatinib, a dual tyrosine-kinase inhibitor of EGFR and HER2 has been approved for the treatment of patients with HER2-positive metastatic breast cancer patients progressing during trastuzumab-based therapy (Geyer *et al*, 2006). Due to the fact that lapatinib is a small molecule, it may penetrate the blood – brain barrier and exhibit prophylactic as well as therapeutic effects. Indeed, Lin *et al* (2008) could demonstrate evidence of modest activity of lapatinib as single agent in 242 patients pretreated with trastuzumab and with progressive CNS metastases after radiotherapy (RR in brain 6%). In the lapatinib plus capecitabine extension phase of that study, a 20% response rate within the CNS was observed (Lin *et al*, 2009). In line with those data, a study recently published even suggested a beneficial impact of lapatinib-based therapy on OS: Significantly longer OS was observed in 30 patients receiving lapatinib and capecitabine after diagnosis of BM as compared with a similar population receiving trastuzumab-based therapies alone (27.9 months *vs* 16.7 months; *P*=0.01) (Metro *et al*, 2011). In that trial, however, not all patients received local treatment for CNS lesions; furthermore, no correction for potential confounders of survival such as KPS or number of BM was performed.

In general, our data fit well into the spectrum of previous studies: patients receiving ongoing systemic therapy showed a marked increase in survival over patients without any further systemic treatment. Patients receiving trastuzumab after completion of local therapy had a median OS of 13 months, which was significantly longer than with chemotherapy alone. In patients receiving lapatinib-based therapy, median OS was still not reached after 24 months of follow-up. This resulted in a significant prolongation of survival over trastuzumab-based therapy alone (*P*=0.002). To correct for other factors significantly associated with OS in the univariate analysis, a Cox proportional hazard models was applied. In that analysis, additional treatment with lapatinib retained significance as predictor of superior outcome (HR for death 0.279; 95% CI: 0.1–0.76; *P*=0.012). Presence of one to three BM was also significantly associated with improved outcome (HR 0.152; 95% CI: 0.06–0.41; *P*<0.001). In line with our observation, Lee *et al* (2008) reported that after completion of local therapy for CNS metastases, ongoing chemotherapy, performance status as well as the number of BM were independent predictors of survival. Similar results were reported by Lentzsch *et al* (1999). In difference to those studies, however, no significant impact of performance status was observed in our analysis. Two facts may add to this observation: first, this was a relatively homogenous population of patients with good performance status, as only 18 out of 80 patients had KPS <80 (22.5%). Furthermore, we suggest that active systemic treatment even in the presence of BM may improve outcome to a point, which reduces the impact of baseline performance status on survival. Indeed, in the overall population of 80 patients, high KPS retained statistical significance as predictor of survival (KPS >70: HR 0.4; 95% CI: 0.23–0.72; *P*=0.002).

Our study is clearly limited by its retrospective nature and single institutional design. However, it represents a relatively homogeneous group of patients presenting with KPS ⩾70, all of whom received optimal local therapy for BM. The imbalance in the percentage of patients treated with radiosurgery or boost irradiation alone is not expected to impact upon survival (Aoyama *et al*, 2006).

In conclusion, our results indicate a significant improvement of OS in patients with BM from HER2-positive breast cancer when treated in addition with an anti-HER2 agent, especially lapatinib. Impact of lapatinib retained statistical significance after correction for other potential confounders of OS, such as presence of visceral metastases, number of extracranial metastatic sites, KPS and number of BM.

## Figures and Tables

**Table 1:** Patient characteristics (total patient population)

**Characteristics**	**Patients**
Entered	*n*=80
Median age at diagnosis of BM (years) (range)	53 (28–77)
Patients ⩾65 years	*n*=14 (17.5%)
Karnofsky Performance Score (median)	80
Patients KPS >70	*n*=62 (77.5%)
Hormone receptor positive	38 (47.5%)
HER2 positive	80 (100%)
Grading 3	58 (72.5%)
Invasive ductal carcinoma	66 (82.5%)

*Stage at primary diagnosis*	
Localised	68 (85%)
Metastatic	12 (15%)
Visceral disease	56 (70%)
>2 Metastatic sites outside the CNS	39 (48.8%)
Only BM	9 (11.3%)
1–3 Brain metastases	40 (50%)
Development of BM in <12 months	23 (28.8%)
Adjuvant chemotherapy	64 (80%)
Adjuvant endocrine therapy	28 (35%)
Palliative chemotherapy	60 (75%)
Palliative endocrine therapy	19 (23.8%)
Trastuzumab before diagnosis of BM	57 (71.3%)
Duration (months) (range)	12.5 (3–57)

*Time to diagnosis of BM (from primary breast cancer diagnosis)*	
Median (months) (range)	36 (0–254)

*Time to diagnosis of BM (from diagnosis of metastatic disease)*	
Median (months) (range)	17 (0–170)
Radiosurgery or boost irradiation without WBRT	7 (8.8%)

*Systemic treatment after local therapy for BM*	
Group A (further anti-HER2 therapy after diagnosis of BM)	43 (53.8%)
Trastuzumab plus/minus chemotherapy	28 (35%)
Lapatinib plus/minus trastuzumab plus/minus chemo	15 (18.8%)
Group B (no anti-HER2 therapy after diagnosis of BM)	37 (46.3%)
No further treatment	28 (35%)
Chemotherapy only	9 (11.3%)

*Duration of trastuzumab treatment after local therapy for BM*	
Months (median) (range)	8.5 (2–37)

*Duration of lapatinib treatment after local therapy for BM*	
Months (median) (range)	8 (2–23)

Abbreviations: BM=brain metastases; CNS=central nervous system; WBRT=whole brain radiotherapy; KPS=Karnofsky Performance Score.

**Table 2:** Patient characteristics (separated for groups A and B)

**Characteristics**	**Group A**	**Group B**	***P* a**
Entered	*n*=43	*n*=37	
Median age at diagnosis of BM (years)	51 (34–74)	56 (28–81)	
Patients ⩾65 years	6 (14%)	8 (21.6%)	NS
Karnofsky Performance Score (median)	80	80	
Patients KPS >70 (%)	34 (79%)	28 (75.7%)	NS
Hormone receptor positive	22 (51.2%)	16 (43.2%)	NS
HER2 positive	43 (100%)	37 (100%)	NS
Grading 3	30 (69.8%)	28 (75.7%)	NS
Invasive ductal carcinoma	35 (81.4%)	31 (83.8%)	NS

*Stage at primary diagnosis*			
Localised	35 (81.4%)	33 (89.2%)	NS
Metastatic	8 (18.6%)	4 (10.8%)	NS
Visceral disease	30 (69.8%)	26 (70.3%)	NS
>2 Metastatic sites outside the CNS	23 (53.5%)	16 (43.2%)	NS
Only BM	3 (7%)	6 (16.2%)	NS
1–3 Brain metastases	23 (53.5%)	17 (45.9%)	NS
Development of BM in <12 months	14 (32.6%)	9 (24.3%)	NS
Radiosurgery or boost without WBRT	6 (14%)	1 (2.7%)	NS

Abbreviations: BM=brain metastases; CNS=central nervous system; WBRT=whole brain radiotherapy; KPS=Karnofsky Performance Score.

a Fisher's exact test.

**Table 3:** Results (Cox proportional hazards model)^a^

	**Total population**			**Patients receiving HER2-targeted therapy after completion of local treatment**		
	***n*=80 (groups A and B)**			***n*=43 (group A)**		
	**HR**	**95% CI**	***P***	**HR**	**95% CI**	***P***
Hormone receptor-positive disease	1.036	0.6–1.78	NS	—	—	—
Visceral metastases	1.727	0.95–3.15	NS	2.177	0.72–6.62	NS
>2 Metastatic sites outside CNS	—	—	—	1.369	0.56–3.43	NS
1–3 Brain metastases	0.322	0.18–0.58	<0.001	0.152	0.06–0.41	<0.001
Diagnosis of brain metastases <12 months	1.293	0.79–2.46	NS	—	—	—
KPS >70	0.404	0.23–0.72	0.002	1.974	0.8–4.86	NS
HER2-targeted therapy after completion of local treatment	0.293	0.16–0.54	<0.001	—	—	—
Lapatinib plus/minus trastuzumab plus/minus chemotherapy after completion of local treatment	—	—	—	0.279	0.1–0.76	0.012

Abbreviations: HR=hazard ratio; CNS=central nervous system; KPS=Karnofsky Performance Score; CI=confidence interval.

a HRs, CIs, and *P*-values are only provided for variables significantly associated with overall survival in the univariate analysis and were therefore included into the Cox Regression Model.
